# In-reply to the comment by Poling et al

**DOI:** 10.1186/s40981-023-00681-1

**Published:** 2024-02-03

**Authors:** Kyosuke Takahashi, Kotaro Sakurai, Izumi Hamaya

**Affiliations:** 1 Department of Anesthesiology and Critical Care Medicine, Jichi Medical University Saitama Medical Center, 1-847 Amanumacho, Omiya-Ku, Saitama, 330-0834 Japan; 2 Department of Anesthesia and Critical Care, Kawasaki Municipal Hospital, 12-1 Shinkawa-Dori, Kawasaki, 210-0013 Japan; 3 Department of Anesthesiology, Kyoto University Hospital, 54 Hogoin-Kawahara-Cho, Kyoto, 606-8507 Japan; 4 Department of Cardiac Anesthesia, Saitama Children’s Medical Center, 1-2 Shin-Toshin, Saitama, 330-8777 Japan

To the editor

We thank Poling et al. for their interest in our report [1]. They raised the importance of a new disease concept and optimal management according to the diagnosis.

As they pointed out, the diagnosis of Freeman-Sheldon syndrome (FSS) (What they call Freeman-Burian syndrome [FBS]) is difficult due to similar physical characteristics between FSS/FBS and Sheldon-Hall syndrome. We think our case was not typical of FSS/FBS. Although she had multiple arthrogryposes and prominent nasolabial folds, microstomia was not severe, midface hypoplasia was mild, and tracheal intubation was easy during anesthesia. However, this does not exclude the diagnosis of FSS/FBS.

The association between distal arthrogryposis and malignant hyperthermia remains uncertain. An observational study that investigated 73 individuals referred with the diagnosis of FSS revealed that 3 out of 10 patients developed malignant hyperthermia when they had surgery [2]. On the other hand, a study reported no association between malignant hyperthermia and distal arthrogryposis [3]. Considering the mixed findings of past studies, it is still safe to avoid inhalation anesthetics because we have many alternative options without them. Hence, our strategy using propofol, opioids, and dexmedetomidine was a reasonable choice for the patient who underwent cardiac surgery. Further studies with large sample sizes are needed to determine the association between malignant hyperthermia and this syndrome. Until this question is resolved, providing malignant-hyperthermia-safe anesthesia for patients with FSS/FBS is warranted.

## Data Availability

Not applicable.

## Abbreviations

FBS: Freeman Burian Syndrome
FSS: Freeman Sheldon Syndrome
